# Application of the Surgical APGAR Score to Predict Intensive Care Unit Admission and Post-Operative Outcomes in Cesarean Hysterectomy for Placenta Accreta Spectrum

**DOI:** 10.3390/medicina61122139

**Published:** 2025-11-30

**Authors:** Emily Root, Jacqueline Curbelo, Patrick Ramsey, Jessian L. Munoz

**Affiliations:** 1 Division of Maternal Fetal Medicine and Perinatal Surgery, Department of Obstetrics and Gynecology, Baylor College of Medicine, Houston, TX 77030, USA; emily.root@bcm.edu; 2 Department of Obstetrics & Gynecology, University of Texas Health Sciences Center at San Antonio, San Antonio, TX 78229, USA; curbelo@uthscsa.edu; 3 University Health System, Department of Anesthesiology, University of Texas Health Sciences Center at San Antonio, San Antonio, TX 78229, USA; ramseyp@uthscsa.edu

**Keywords:** Placenta Accreta Spectrum, morbidity, risk-reduction

## Abstract

*Background and Objective*: Placenta Accreta Spectrum (PAS) encompasses a continuum of abnormal placentation conditions associated with significant maternal and fetal morbidity. Management of PAS requires coordinated cesarean hysterectomy. Associated morbidities include blood transfusion, coagulopathy, and intensive care unit (ICU) admission. Accurate prediction of ICU admission allows for enhanced multidisciplinary management, coordination of care and utilization of resources. Scoring systems exist in other surgical specialties that can predict the likelihood of ICU admission, but these have not been applied to an obstetric population. The SAS is a 10-point scale that has been validated for the prediction of ICU-level care requirements within 72 h post-operatively in numerous surgical specialties. The purpose of this study was to apply the Surgical APGAR Score (SAS, version 9) to patients undergoing management of PAS to determine if it can predict ICU admission in this population. *Materials and Methods*: This is a case–control study. We retrospectively analyzed 127 cases of pathology-confirmed PAS patients who underwent cesarean hysterectomy in singleton, non-anomalous, viable pregnancies. Our primary outcome was ICU admission. In addition, secondary outcomes included antepartum characteristics, operative time, intraoperative events as well as post-operative complications and total postoperative length of stay. SAS was assigned by extracting estimated blood loss (EBL), and the lowest mean intraoperative heartrate (HR and mean arterial pressure (MAP) from intraoperative documentation. Categorical and continuous factors were summarized using frequencies and percentages or means ± SD or median and range as appropriate. Pearson’s chi-square, Fisher’s exact tests, and Mann–Whitney U and *t*-tests were applied when appropriate. Logistical regression to assess the impact of SAS on ICU admission was performed. *p*-values < 0.05 were considered significant for two-tailed analysis. Statistical analysis was performed using Graphpad software (version 9). *Results*: Fifty-eight patients (45%) were admitted post-operatively to the ICU, while 69 patients (55%) were admitted for routine care to the post-anesthesia care unit. Baseline demographics were similar between groups. Forty-four patients (52%) admitted to the ICU had a SAS score < 4. SAS < 4 was associated with greater blood loss (3000 vs. 2500 mL, *p* = 0.03) and longer operative time (198 vs. 175 min, *p* = 0.03). Logistic regression analysis of SAS score and ICU admission revealed a low predictive value (OR 2.28, AUC = 0.599). *Conclusions:* The SAS system is a poor tool for the prediction of ICU admission in patients with PAS undergoing cesarean hysterectomy. A risk calculator that accounts for the unique physiologic changes in pregnancy and high risk for pregnancy is needed.

## 1. Introduction

Placenta Accreta Spectrum (PAS) is a continuum of placental adherence disorders characterized by significant maternal morbidity and mortality. The definition of maternal morbidity is based on ICD-10 codes that correspond to a number of conditions, including but not limited to blood transfusion, coagulopathy, and Intensive Care Unit (ICU) admission [1]. Preventing post-operative morbidity and reducing the rate of maternal mortality is a key public health goal amongst many nations, and is listed as a component of the “2030 Agenda for Sustainable Development,” a World Health Organization initiative to transform health worldwide [2]. According to the WHO report, over 700 women died from preventable causes related to childbirth in 2023; the most common cause of maternal mortality world-wide is postpartum hemorrhage [2]. The estimated rate of PAS has risen from 1 in over 2500 pregnancies forty years ago to 1 in 272 [3]. Maternal morbidity and mortality can occur in cases of PAS due to life-threatening hemorrhage [3]. Preoperative planning can allow providers to have optimal resources prior to the procedure. In fields outside of obstetrics and gynecology as well as when considering best practices in terms of management of PAS, multidisciplinary team management and effective perioperative planning has been shown to be associated with decreased surgical morbidity [4,5]. Unfortunately, unlike in other surgical specialties, there is not a widely used scoring system in the field of obstetrics that can help providers stratify the risk of post-operative morbidity after traditional management of PAS, the cesarean hysterectomy.

The incidence of PAS has increased worldwide, and recommended management in the majority of cases is a scheduled preterm cesarean hysterectomy, prior to the onset of labor, at a multidisciplinary center with experienced providers and an available NICU team [6]. There is not a global consensus on the best method for antepartum screening for severity of disease, and with the rising incidence of PAS cases worldwide it is critical that significant strides are made to reduce the incidence of maternal mortality in PAS cases [7,8]. In fact, population-based studies in the United States, United Kingdom, and from the Nordic Obstetric Surveillance Study (includes Denmark, Iceland, and Norway) have shown that PAS remains undiagnosed prior to delivery in around 50% of cases [9,10,11]. Ultrasound is the recommended first line modality for diagnosing PAS [3]. The etiology of the missed cases of PAS prior to delivery is multifactorial. Ultrasound is a modality that is operator dependent, there can significant variability in image acquisition among sonographers and machines, the ultrasound markers of PAS can be subjective and may be difficult to interpret in settings with low exposure to PAS, and other societal limitations in the United States including access to healthcare [12]. In an effort to identify more cases of PAS, other imaging modalities such as MRI have been evaluated, but in a systematic review and meta-analysis it was found that there was no statistically significant differences between modalities [13]. Despite advances in technology and the strides made in utilizing technology such as magnetic resonance imaging (MRI) for identifying PAS and efforts to standardize imaging protocols using ultrasound, a large number of cases are still not identified, and these cases do not benefit from the protective effects of preoperative planning.

Since we are lacking tools to accurately predict which patients are more likely to experience post-operative morbidity prior to presenting for delivery, utilizing a tool intraoperatively to stratify patient risk could help guide providers in pursuing preventive strategies. A number of scoring systems have been proposed to predict postoperative outcomes in the non-obstetric population including the American Society of Anesthesiologists-Physical Status (ASA-PS), Acute Physiology and Chronic Health Evaluation (APACHE) scoring system, the Physiological and Operative Severity Score for the Enumeration of Mortality and Morbidity (POSSUM), the Simplified Acute Physiology Score (SAPS), and the Surgical Apgar Score (SAS) [14]. The ASA classification system although used in the field of anesthesia for risk stratification, it was not designed to predict postoperative morbidity, it is instead more predictive of the patient’s risk for intraoperative complications. POSSUM has been found to not be applicable in trauma patients, and SAPS and APACHE are more reliable in predictive outcomes in medical patients versus surgical [14]. The SAS was developed in 2007 to predict post-operative morbidity following colectomy, and since then has been validated by prospective studies in urologic, orthopedic, neurologic surgery, and in cases of emergency laparotomy [4,14,15]. To date, SAS has not been applied to obstetric surgery and specifically has not been applied in cases of PAS.

Given the high risk of post-operative complications in cesarean hysterectomy cases for management of PAS, including profound blood loss, transfusion of blood products, and ICU admission, our objective was to retrospectively apply the SAS system to our PAS hysterectomy database and assess validity in this cohort.

## 2. Materials and Methods

This is a case–control study. We conducted a retrospective chart review of women who presented to the University Hospital System/University of Texas Center for Placenta Accreta Spectrum program between January 2005 and December 2021 [4]. Institutional review board (IRB) approval was obtained from the University of Texas Health San Antonio and University Hospital System prior to obtaining patient information from electronic medical records. Inclusion criteria included maternal age between 18 and 55 years with a viable pregnancy and antenatal suspicion for PAS. Risk for PAS was determined by sonographic findings concerning for PAS, especially in the context of prior uterine surgery, as well as the history-based risk which applied to patients with a placenta previa. Sonographic findings suspicious for PAS as per the Society for Maternal Fetal Medicine PAS ultrasound marker task force [12], MRI imaging with concern for PAS per the radiologist report. An a priori risk score was also considered based on maternal comorbidities such as presence of previa and the patient’s history of cesarean section. Final patient inclusion was dependent on pathology confirmation of PAS by a board-certified pathologist with extensive gynecologic experience. Exclusion criteria were the following: incomplete records, fetal death, gestational age < 20 weeks, conservative management of PAS, and associated fetal congenital anomalies.

The University Hospital System/University of Texas Center for Placenta Accreta Spectrum to provide a protocol-driven multidisciplinary approach to the management of PAS cases in south central Texas. Within the program, patients were managed by a multidisciplinary team as described above. Strengthening the reporting of observational studies in epidemiology (STROBE) guidelines were followed throughout this study.

Data were collected and stored using a REDCap electronic data capture tool hosted at the University of Texas Health San Antonio. REDCap (Research Electronic Data Capture, Vanderbilt University, Nashville, TN, USA) is a secure, web-based application designed to support data capture for research studies [5].

### 2.1. Study Outcomes

Maternal demographic, prenatal, and operative information was obtained retrospectively from electronic medical records. Our primary outcome was ICU admission. In addition, secondary outcomes included antepartum characteristics, operative time, intraoperative events as well as post-operative complications and total postoperative length of stay.

SAS was assigned by computing estimated blood loss (EBL), and the lowest mean intraoperative heartrate (HR) and mean arterial pressure (MAP). These values were extracted from the anesthesia intraoperative recording sheet retrospectively. While the previously described Surgical Apgar Score (SAS) has three categories (0–4, 5–8, 9–10), all patients were categorized as 0–4 or 5–8 in binary fashion due to the impact of EBL in cesarean hysterectomy cases [15]. SAS scores closer to 0 points are associated with poor outcomes, the maximum number of points received with this scoring system is 10 points. Zero points are associated with an EBL greater than one liter, the average lowest intraoperative MAP less than 40, and lowest heart rate greater than 85.

### 2.2. Statistical Analysis

Normal distribution was determined by Shapiro–Wilk test results greater than 0.05. Pearson’s chi-square (χ2), Fisher’s exact tests, and Mann–Whitney U and *t*-tests were applied when appropriate. Categorical factors were summarized using frequencies and percentages, while continuous measures summaries used means ± SD or median and range as appropriate. Logistical regression was utilized to assess the impact of SAS as a binary variable and ICU admission. *p*-values < 0.05 were considered significant for two-tailed analysis. Statistical analysis was performed using Graphpad software (version 9).

## 3. Results

**Figure 1:**
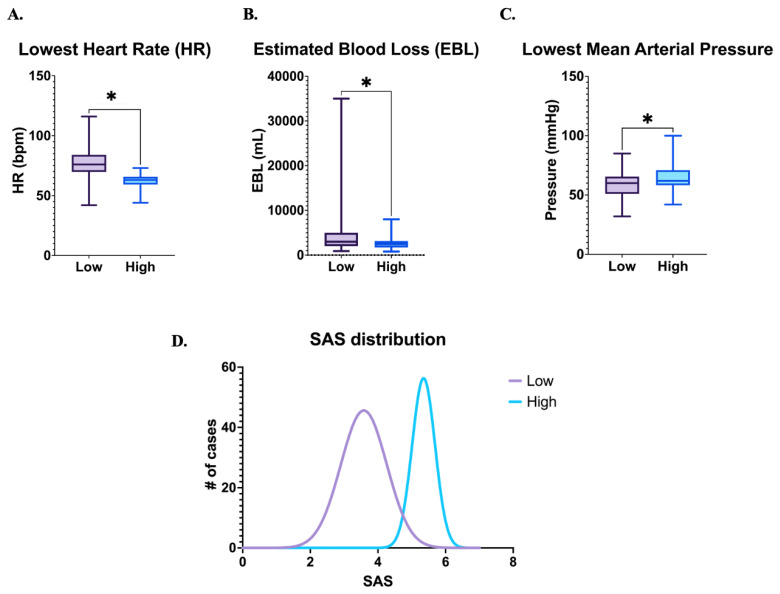
SAS scoring and distribution. # number, * *p* < 0.05.

From January 2005 to December 2021, 156 cases of PAS were managed by the multidisciplinary team at our institution. Of these, 127 met inclusion and exclusion criteria as described previously. Intraoperative records for all patients were available and reviewed. 84 patients (66.1%) had a “low” SAS score of 0–4 and 43 (33.9%) had a “high” SAS score of 5–8. SAS component confirmation showed statistically significant differences in heart rate, estimated blood loss, and mean arterial pressure (Figure 1A–C). SAS distribution among our patient population demonstrated that the majority of patients had a raw score of 4 or 5 (Figure 1D).

Analysis of the study population noted significantly lower gestational age at delivery with SAS 0–4 (34 vs. 35 weeks, *p* < 0.001). All other demographic parameters with equivalent between compared groups (Table 1).

Pregnancy complication such as episodes of vaginal bleeding, preterm labor, fetal growth restriction, and hypertensive disorders of pregnancy were analyzed and noted to not be significantly different (Table 2).

Intraoperatively, patients with a SAS of 0–4 were more likely to exhibit a number of maternal morbidities including longer operative time (198 vs. 175 min, *p* = 0.03), longer exposure to general anesthesia (216 vs. 150 min, *p* = 0.02), less utilization of intentional cystotomies (0 vs. 9.3%, *p* = 0.01), more likely to be admitted to the ICU (52 vs. 33%, *p* = 0.03), and longer ICU admissions (1 vs. 0 days, *p* = 0.02) (Table 3).

**Figure 2:**
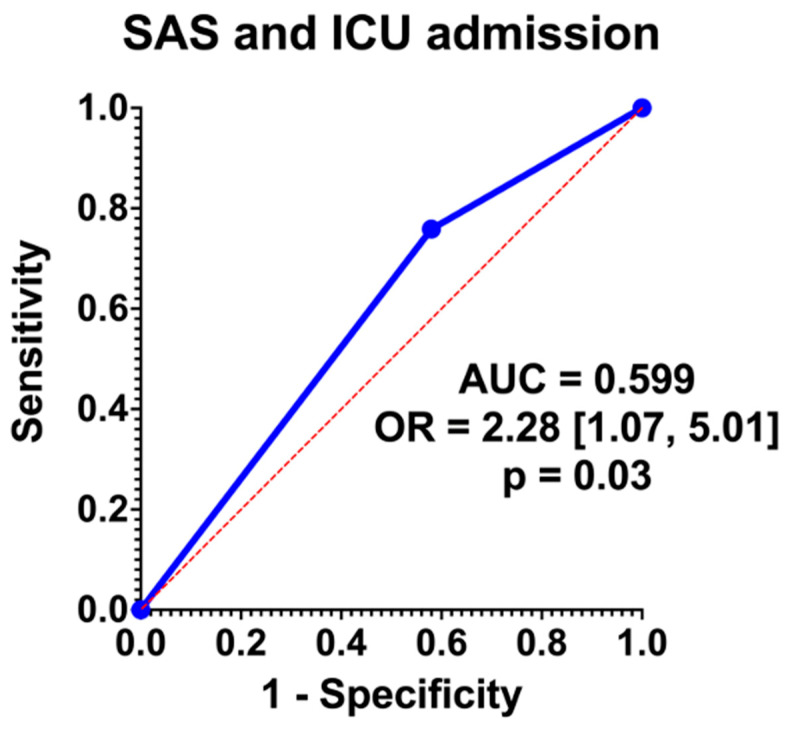
Regression analysis.

Logistic regression analysis showed that SAS 0–4 had an OR = 2.28 [95% CI 1.07, 5.01]. Graphical representation is noted in Figure 2. The area under the curve (AUC) was 0.599.

## 4. Discussion

Patients in our cohort with PAS undergoing a cesarean hysterectomy with a low SAS were more likely to be admitted to the ICU, although the area under the curve of SAS for ICU prediction was 0.599 (Figure 2). While this represents overall poor predictive capabilities, this presents an opportunity for optimization and further research. In terms of prenatal characteristics, lower gestational ages were associated with lower SAS scores (34 vs. 35 weeks, *p* = 0.001), but antenatal factors associated with complex pregnancies were not significantly associated with SAS (Table 2). Intraoperatively, lower SAS scores were significantly associated with longer operative times, larger blood loss, greater anesthesia exposure, and fewer intentional cystotomies (Table 3). Although there was a distinction in terms of gestational age and SAS score, this is likely more a reflection of the extent of disease and the need for an urgent or emergent delivery. This same principle applies to the reasoning behind longer operative time, increased EBL, and anesthesia exposure were associated with a lower SAS score and ultimately ICU admission. Of note, the finding of decreased intentional cystotomy in lower SAS cases may be a reflection of the small sample size, since cases where cystotomy is required due to extensive disease would be more likely to be associated with ICU admission. Of note, there is a higher rate of incidental cystotomy in the group with lower SAS, but this finding did not reach statistical significance. This is the first study to the authors’ knowledge that has applied the SAS to an obstetric population, and in particular, in the management of PAS. Further analysis is necessary to determine if this scoring system is feasible in predicting ICU admission in a prospective study.

The SAS tool has performed better in other specialties (trauma setting for emergent laparotomy AUC 0.712, esophagectomy AUC 0.62, laparotomy AUC 0.75, high risk intra-abdominal surgery 0.63) [4,16,17,18]. Despite the appeal of this system, the pregnant population has unique characteristics that hinder the ability to use a scoring system that is generalized for a broad patient population.

The SAS system would appear to be an ideal tool for assessing post-operative risks in obstetric patients undergoing cesarean hysterectomy. The ease of a simple 3-point system allows for quick and timely application for both the utilization of hospital resources and disposition for optimal patient care. Due to the impact of EBL (average EBL 2–4 L) in our group, we transitioned the SAS into a binary system. During a cesarean delivery a hysterotomy must be made without blocking blood flow to the infant prior to delivery. This contrasts with the concepts of isolating the blood supply, ligating, and transecting when dissecting an organ as is seen in surgical technique in other specialties. In the third trimester the uteroplacental blood flow can reach around 1000 cc per minute at term, which accounts for 20% of the maternal cardiac output [19]. This adaption in pregnancy is necessary to support fetal growth but may also be associated with increased risks of hemorrhage. In cases of PAS, there are multiple approaches to management that extend beyond the purposes of this paper. Blood loss during cesarean hysterectomy can be even further compounded due to disrupted uterine and pelvic vessels, placental separation and uterine dehiscence, and the surgical field is made more complex due to the increased vascularity and the dilated collateral blood supply that is unique to pregnancy [20]. The large volume of blood loss that can be experienced in PAS cases during cesarean hysterectomy likely contribute to the inability of the SAS system to predict ICU admission.

In addition, maternal physiologic changes may also contribute to the limitations of the SAS in predicting post-operative morbidity in our pregnant population with PAS. Pregnancy is associated with significant cardiovascular physiologic changes that are needed in order to adequately supply both the mother and fetus from a metabolic standpoint, but also provide adequate perfusion [21]. Maternal heart rate increases by up to 20 bpm by the third trimester, which is a 20% increase over baseline [21]. The SAS system only gives patients a point under the heart rate category once the heart rate is less than 85, but tachycardia in pregnancy is defined as a heart rate greater than 100 bpm [22].

The average MAP in the third trimester is around 80 mmHg, and in the immediate postpartum period can range from 70–100 mmHg [23,24]. In known PAS cases the goal is to avoid disrupting the placenta in order to reduce uterine contraction and bleeding from the morbidly adherent placenta. In the immediate postpartum period as the placenta attempts to shear from the uterus a dynamic set of vascular changes occur including decrease in blood volume, reduced vascular contractility, venous stasis particularly in the left pelvic veins, and a slow return of MAP which is still influenced by elevated cardiac output and overall vasodilation [21]. In a standard delivery, as the placenta is delivered there is a resulting autotransfusion, and the cardiac output increases, in some cases to 80% above pre-pregnancy values [23]. Beyond the expected maternal physiologic changes that occur intraoperatively, there are iatrogenic causes to changes in maternal MAP. Regional anesthesia, via either epidural or spinal analgesia, can also have vasodilatory effects, although these effects are less likely to be as important postpartum. Given the physiologic and iatrogenic impacts on MAP, using the SAS may not be as predictive.

Maternal mean arterial pressure describes the average pressure in arteries during the cardiac cycle and reflects perfusion. There are dynamic changes that occur to blood pressure during pregnancy due to the influence of increased plasma volume, systemic vasodilation, and increasing cardiac output. In the setting of hemorrhage, pregnant patients in the third trimester have increased plasma volume (around 50% above baseline), and compensatory mechanisms that can mask the effects of a massive hemorrhage until a significant blood volume has been lost (around 25% of the patient’s blood volume, or around 1000–1500 cc) [25].

Further research optimizing the scoring system for large volume blood loss and pregnancy may result in a system, which may allow for optimized prediction and resource allocation intraoperatively during cesarean hysterectomy cases. Additionally, continued work is needed to improve our abilities to predict PAS prior to delivery in order to optimize the patient prior to surgery and benefit from the improved outcomes seen in multidisciplinary planned cases [5].

The present study has several notable strengths and weaknesses. As a single-center study, this allows for uniformity of patient care with a multidisciplinary team. Yet, data has shown referral centers and multidisciplinary teams tend to have greater degrees of pathology and in turn, morbidity [9]. For outcomes evaluated at a large referral center there is the risk of referral bias since the patients represented in the sample are not representative of the overall population. Thus, data may not be applicable to smaller hospitals or those in rural settings with resource limitations. This study also has limitations due to the retrospective nature of the study design, the sample size, and the possible variability in surgical techniques or experience level of the surgeons.

## 5. Conclusions

The SAS system should not be applied in pregnant patients undergoing cesarean hysterectomy for PAS management. A risk calculator that accounts for the unique physiologic changes in pregnancy, the dynamic mechanism for compensation during blood loss, and the large volume EBL would be ideal for this population. Risk calculators have proven to be invaluable in improving outcomes in other populations. Emphasis on creating a tool for an obstetric population may be the key to reducing maternal morbidity worldwide.

## Figures and Tables

**Table 1:** Patient demographics.

Factor	SAS 0–4 (n = 84)	SAS 5–8 (n = 43)	*p*-Value
**Age**	31.2 ± 5.5	30.84 ± 4.4	0.68
**BMI**	32.8 ± 6.2	33.4 ± 6.3	0.61
**Gravity**	5 [3, 6]	5 [4, 5]	0.99
**Parity**	3 [2, 4]	3 [2, 4]	0.86
**History of CD**	77 (92)	39 (91)	1.0
**Tertiary referral**	64	29	0.39
**GA at delivery**	34 [32, 35]	35 [34, 37]	0.001 *
**PAS by Ultrasound**			
**Previa**	34 (40.1)	16 (37.2)	0.84
**Accreta**	29 (34.5)	19 (44.2)	0.33
**Increta**	2 (2.4)	0 (0)	0.55
**Percreta**	19 (22.6)	8 (18.6)	0.65
**Diabetes**	5 (5.9)	5 (11.6)	0.30
**Hypertension**	8 (9.5)	4 (9.3)	1.0
**Anemia**	32 (38)	18 (41.9)	0.70
**Emergent delivery**	28 (33.3)	12 (27.9)	0.68
**Public insurance**	64 (76.2)	31 (72.1)	0.66

Values presented as Mean ± SD, Median [P25, P75], or N (column %), BMI = body mass index, CD = cesarean delivery, GA = Gestational Age, * *p*-value < 0.05.

**Table 2:** Pregnancy complications.

Factor	SAS 0–4 (n = 84)	SAS 5–8 (n = 43)	*p*-Value
**Antepartum admission**	58 (69.0)	25 (58.1)	0.24
**Antepartum LOS**	2 [0, 8]	1 [0, 3]	0.14
**Vaginal bleeding**			
**×1**	13 (15.5)	5 (11.6)	0.78
**×2**	13 (15.5)	6 (13.9)	1.0
**>2**	11 (13.1)	4 (9.3)	0.77
**PPROM**	5 (5.9)	4 (9.3)	0.48
**Preterm labor**	6 (7.1)	1 (2.3)	0.42
**FGR**	2 (2.4)	1 (2.3)	1.0
**Gestational hypertension**	3 (3.6)	3 (6.9)	0.40
**Pre-eclampsia without severe features**	0 (0)	2 (4.7)	0.11
**Pre-eclampsia with severe features**	3 (3.6)	3 (6.9)	0.40
**Gestational diabetes**	15 (17.9)	9 (20.9)	0.81

Variables presented: LOS = length of stay, PPROM = preterm prelabor rupture of membranes, FGR = fetal growth restriction, *p* < 0.05.

**Table 3:** Operative characteristics.

Factor	SAS 0–4 (n = 84)	SAS 5–8 (n = 43)	*p*-Value
**Admission hgb (g/dL)**			0.84
**ASA Classification**	11.03 ± 2.48	11.12 ± 1.41	
**I**			0.54
**II**	2 (2.4)	0 (0)	0.76
**III**	8 (9.5)	5 (11.6)	0.42
**IV–V**	56 (66.7)	32 (74.4)	0.47
**Operative time (min)**	17 (20.2)	6 (13.9)	0.03 *
**Urinary stent placement**	198 [154, 310]	175 [124, 247]	1.0
**EBL (mL)**	36 (42.8)	19 (44.1)	0.02 *
**GETA exposure (min)**	3000 [2000, 5000]	2500 [1700, 3150]	0.004 *
**Transfusion (any)**	216 [119, 381]	150 [0, 223]	0.23
**Whole blood**	77 (91.7)	36 (83.7)	1.0
**Red blood cells**	13 (15.5)	6 (13.9)	0.81
**Platelets**	69 (82.1)	34 (79.1)	0.84
**Fresh frozen plasma**	28 (33.3)	13 (30.2)	0.26
**Cryoprecipitate**	50 (59.5)	21 (48.8)	0.14
**GU injury**	12 (14.3)	2 (4.7)	
**Intentional cystotomy**			0.01 *
**Incidental cystotomy**	0 (0)	4 (9.3)	0.36
**Ureteral injury**	20 (23.8)	7 (16.3)	0.66
**PAS by pathology**	4 (4.7)	1 (2.3)	
**Accreta**			0.13
**Increta**	17 (20.2)	14 (32.6)	0.83
**Percreta**	24 (28.6)	13 (30.2)	0.19
**ICU admission**	43 (51.2)	16 (37.2)	0.03 *
**ICU LOS**	44 (52)	14 (33)	0.02 *
**Post-operative LOS**	1 [0, 1]	0 [0, 1]	0.27

Hgb = hemoglobin, EBL = Estimated blood loss, GETA = general endotracheal anesthesia, GU = Genitourinary, PAS = Placenta accreta spectrum, ICU = intensive care unit, LOS = length of stay, ASA = American Society of Anesthesiologists. Values presented as Mean ± SD, Median [P25, P75] or N (column %). * *p* < 0.05.

## Data Availability

The data presented in this study are available on request from the corresponding author due to patient privacy.

## Abbreviations

The following abbreviations are used in this manuscript:

SAS	Surgical apgar score
ICU	Intensive care unit
PAS	Placenta accreta spectrum
WHO	World Health Organization
IRB	Institutional Review Board
STROBE	Strengthening the reporting of observational studies in epidemiology
ASA-PS	American Society of Anesthesiologists-Physical Status
APACHE	Acute Physiology and Chronic Health Evaluation
POSSUM	Physiological and Operative Severity Score for the Enumeration of Mortality and Morbidity
SAPS	Simplified Acute Physiology Score
MRI	Magnetic resonance imaging
MAP	Mean arterial pressure
BMI	Body mass index
CD	Cesarean delivery
GA	Gestational age
LOS	Length of stay
PPROM	Preterm pre-labor rupture of membranes
FGR	Fetal growth restriction
GD	Gestational diabetes
Hgb	Hemoglobin
EBL	Estimated blood loss
GETA	General endotracheal anesthesia
GU	Genitourinary
AUC	Area under the curve
